# Associations between diet quality, blood pressure, and glucose levels among pregnant women in the Asian megacity of Jakarta

**DOI:** 10.1371/journal.pone.0242150

**Published:** 2020-11-25

**Authors:** Deviana A. S. Siregar, Davrina Rianda, Rima Irwinda, Annisa Dwi Utami, Hanifa Hanifa, Anuraj H. Shankar, Rina Agustina

**Affiliations:** 1 Department of Nutrition, Faculty of Medicine, Universitas Indonesia, Dr. Cipto Mangunkusumo General Hospital, Jakarta, Indonesia; 2 Human Nutrition Research Center, Indonesian Medical Education and Research Institute, Faculty of Medicine, Universitas Indonesia, Jakarta, Indonesia; 3 Department of Obstetrics and Gynecology, Faculty of Medicine, Universitas Indonesia, Dr. Cipto Mangunkusumo General Hospital, Jakarta, Indonesia; 4 Centre for Tropical Medicine and Global Health, Nuffield Department of Medicine, University of Oxford, Oxford, United Kingdom; 5 Eijkman-Oxford Clinical Research Unit, Eijkman Institute for Molecular Biology, Jakarta, Indonesia; University of Mississippi Medical Center, UNITED STATES

## Abstract

**Background:**

The prevalence of gestational hypertension and diabetes in pregnancy is increasing worldwide. Diet is a modifiable factor that may influence these conditions, but few studies have examined the association between diet quality and blood pressure and glucose profiles among pregnant women. Data are especially scarce for women in low- and middle-income countries (LMICs), where 90% of global pregnancies occur, and in urban settings. We, therefore, assessed these associations among 174 pregnant women in the Asian megacity of Jakarta in a cross-sectional study of the Brain Probiotic and LC-PUFA Intervention for Optimum Early Life (BRAVE) project.

**Methods:**

Trained field-enumerators collected socio-demographic characteristics, measured Mid-Upper Arm Circumference (MUAC), and assessed diet by two 24-hour recalls, which were used to calculate the Alternate Healthy Eating Index for Pregnancy (AHEI-P). Blood pressure was measured by automated sphygmomanometer, and fasting blood glucose by capillary glucometer. General linear models were used to identify associations.

**Results:**

The median AHEI-P score was 47.4 (IQR 19.1–76.6). The middle tertile of the AHEI-P score (39.59–56.58) was associated with a 0.4 SD (standardized effect size, 95% CI -0.7 to -0.06; p = 0.02) lower diastolic blood pressure compared with the lowest tertile (<39.59), after adjustment for level of education, smoking status, MUAC, gestational age, history of hypertension, and family history of hypertension. However, no associations were found between the AHEI-P score and systolic blood pressure and blood glucose.

**Conclusion:**

Higher diet quality was associated with lower diastolic blood pressure among pregnant women in an urban LMIC community, but not with systolic blood pressure and blood glucose. A behavioral change intervention trial would be warranted to confirm the influence of diet quality on blood pressure and glucose levels and among pregnant women, and even before pregnancy.

## Introduction

The prevalence of Gestational Diabetes Mellitus (GDM) is increasing worldwide, and ranges across countries from 1–20%, with 16.9% of all pregnancies being affected. The highest prevalence of GDM is in South-East Asia, with an estimated 25% of pregnancies affected [1]. In 2013, the World Health Organization (WHO) reported the prevalence of GDM was 7.48% in high-income groups in both East and South-East Asia but was 17.6% in the lower- and upper-middle groups [2]. In addition, maternal hypertensive disorders (MHD) affect 10% of pregnancies worldwide [3]. These non-communicable diseases (NCDs) may affect pregnancy outcomes and maternal and infant health in later life.

Women with GDM are 2.43 times more likely to have macrosomic newborns, who are in turn 11.7 times more likely to be hyperglycemic at birth. Women with GDM also have an elevated risk of impaired glucose tolerance and type 2 diabetes in the years following pregnancy. Moreover, their children are more likely to be obese and have impaired glucose tolerance and diabetes in early adulthood [4, 5]. Likewise, women with MHD have increased risk of adverse pregnancy outcomes, including low birth weight, stillbirth, and newborns with the low Apgar score at 5 minutes [6].

Healthy eating habits contribute to the prevention of multiple pregnancy-related complications [7]. Indeed, dietary intake is suggested to be a substantial modifiable risk for GDM and MHD [8]. In the general population, dietary fat intake, both quantity, and quality affect glucose tolerance and insulin sensitivity [9]. WHO and Food and Agriculture Organization (FAO) report that proper intake of dietary fiber, fruits, and vegetables helps prevent obesity, diabetes, and cardiovascular disease [10]. Moreover, diets that score high on the Alternate Healthy Eating Index (AHEI) are associated with a lower risk of cardiovascular disease and type 2 diabetes in women [8, 11, 12]. However, few studies investigated the link between AHEI in pregnancy and GDM or MHD, and fewer still have assessed this in low- and middle-income countries (LMICs), where 90% of global pregnancies occur, or in urban settings where dietary habits are rapidly changing.

Considering the importance of diet quality during pregnancy as a modifiable contributor to GDM and MHD in urban LMIC settings, we aimed to evaluate the association between diet quality using the Alternate Healthy Eating Index for Pregnancy (AHEI-P) and blood glucose and blood pressure among pregnant women in Jakarta, Indonesia, an Asian megacity. Moreover, we focused our study on the second-trimester of pregnancy as this corresponds to the onset of substantial metabolic and physiological changes [13, 14].

## Material and methods

### Study design and participants

This cross-sectional study is part of the Brain Probiotic and LC-PUFA Intervention for Optimum Early Life (BRAVE) project (NCT 03851120). Study participants were pregnant women recruited during antenatal care visits in public health centers in three densely populated areas of Central, South, and North Jakarta. Centers were selected based on the higher number of pregnant women attending antenatal care sessions. Women were chosen consecutively at presentation based on gestational age in the 2^nd^ trimester. At the first encounter, written informed consent was obtained from the women, and a brief interview was administered regarding maternal characteristics, 24-hour dietary recall, blood pressure and mid-upper arm circumference (MUAC) were assessed. At the second study visit, which occurred at the next available weekend, we administered a second 24-hour dietary recall and fasting blood glucose assessment.

We estimated a minimum sample size of 123 pregnant women was required to detect a Spearman’s correlation of 0.3, assuming a design effect of 1.5 for a normally distributed outcome (power 80%, *α* = 0.05) [15]; assuming a 10% of non-response rate, we aimed to recruit 135 women. From July to August 2019, we enrolled 202 pregnant women. Women with multiple gestations, e.g. twin or triplets, and those with incomplete dietary recall (n = 26) were excluded from the analysis.

### Instrument

A structured questionnaire was used to assess maternal characteristics. Dietary intake was assessed using multiple 24-hour recalls, which were intake from a weekday and a weekend day. To estimate the portions of food intake, we used a food picture book published by the Ministry of Health of the Republic of Indonesia [16]. MUAC measurement was performed by trained enumerators using SECA measuring tape (Medisave UK Ltd., UK). The mean of two measurements were calculated to the nearest 0.1 cm.

### Blood glucose and blood pressure measurement

Fasting capillary blood glucose has been demonstrated to be an acceptable and useful screening test for GDM [17], and was assessed using the Accu-check Performa II (Roche Diabetes Care, Inc., Australia). Pregnant women were required to fast at least 8 hours before the assessment and to avoid high physical activity. Blood pressure was measured using the OMRON HBP-1300 automated sphygmomanometer (Omron Healthcare Co., Ltd., China), which is clinically proven to produce reliable results [18]. The cuff was placed securely around the upper arm at the level of the heart, with the lower arm being passively supported. Readings were recorded from the automated digital display, and the process was repeated after 5 minutes. This was performed during the first encounter at enrolment in the public health center from 09.00 AM– 12.00 PM [18].

### Diet quality assessment (AHEI-P)

AHEI-P was adapted from the AHEI. The AHEI was based on foods and nutrients predictive of chronic diseases [19]. A previous study demonstrated that a higher AHEI score was strongly associated with a lower risk of major chronic disease and type 2 diabetes [8, 12]. AHEI-P is slightly modified for pregnancy and has additional components to reflect the intake of important nutrients during pregnancy: folate, iron, and calcium. For the AHEI-P, there are nine food-based components, including vegetables, fruits, the ratio of white to red meat, fiber, trans fat, the ratio of polyunsaturated (PUFA) to saturated fatty acid (SFA), folate, calcium, and iron from foods. Intake assessment is food-based and does not include vitamins and minerals from supplements or non-food items, and alcohol is excluded as it is not recommended during pregnancy; moreover, consumption of alcohol in Indonesia among pregnant women is very low [18–20]. Poultry and fish are categorized as white meat, whereas beef, or lamb, and processed meats are classified as red meat. Each component contributes 0 to 10 points to measure diet quality on a 90-point scale. A score of 10 indicated the subject met the recommendation, while 0 was the least healthy diet. We used two 24-hours recalls to assess intake. The mean intake of these recalls was calculated according to the serving sizes of each food component. The component score was calculated by multiplying the serving size by the maximum score, then dividing by the recommended serving size per day. The total score of diet quality was obtained by summing across all components scores [20]. After thoroughly re-checking the dietary recall data, we excluded two pregnant women with calculated energy intake <500 kcal and ≥3500 kcal, as these were deemed implausible [21]. For multivariate regression using AHEI-P as a covariate, we recoded into tertiles with the first being the lowest quality and the third the highest.

### Statistical analysis

Data were analyzed using Statistical Product and Service Solutions (SPSS) version 20.0 (IBM, United States). Descriptive statistics were percentages for categorical data, and medians, means and standard deviations for continuous variables. Data of diastolic blood pressure was transformed by replacing the variable by the square of initial data. Normality was assessed using the W/S test. Bivariable analyses of several confounders of diet quality on blood glucose and blood pressure were performed using one-way ANOVA, independent *t-test*, and Pearson’s correlation. Multiple linear regression was used to examine the associations between the diet quality and blood glucose and blood pressure adjusted for potential confounders, i.e. level of education, history of NCDs, smoking status, MUAC, and gestational age. An association with p<0.05 was considered significant [22]. Results of the regression were provided as standardized effect size calculated by dividing B coefficient with the pooled standard deviation.

### Ethical statements

This study was a part of the BRAVE Project with ethical approval from the Ethics Committee of Faculty of Medicine Universitas Indonesia (No. 0071/UN2.F1/ETIK/2018). All subjects who intended to participate signed a written informed consent prior to the start of the study. This study is registered with ClinicalTrials.gov (NCT03851120).

## Results

We included 174 pregnant women in the analysis. Table 1 presents the median values of each component of AHEI-P, and Table 2 shows the socio-demographic characteristics among pregnant women. Most were considered as low-risk pregnancies (76%) based on the age of the respondents (20–34 years old), yet only 39% were nulliparous. About 74% of mothers had finished junior high school, and 32% were employed at the time of assessment. The median diet quality score of the women was only 47.4 (minimum 19.1, maximum 76.6) out of 90. This low score was due to low consumption of fruit, high consumption of SFA compared to PUFA, and low consumption food-based folate indicated by low median scores of 2.2, 2.03, and 3.6, respectively. Moreover, the median scores for vegetables and food-based iron reached only half of the maximum score (5.98 and 5.46), whereas median scores of fiber and calcium consumption were slightly lower (4.15 and 4.65). The mean value of fasting blood glucose was 89.6 mg/dL and ranged from 70 to 159 mg/dL, mean systolic blood pressure was 109 mmHg ranging from 72 to 139 mmHg, and diastolic blood pressure was 69 mmHg ranging from 51 to 98 mmHg.

**Table 1:** Diet quality score among pregnant women recruited in primary health centers of Jakarta (n = 174) [8, 19, 20].

Component	Criteria for maximum score of 10^a^	AHEI-P score Median (25^th^, 75^th^)
Vegetables (servings/d)^b^	≥5	5.98 (3.35, 9.66)
Fruit (servings/d)^c^	≥4	2.20 (0.66, 3.75)
Ratio of white to red meat	≥4	10.0 (7.91, 10.0)
Fiber (g/d)	≥25	4.15 (2.95, 5.90)
Trans fat (% of energy)	≤0.5	10.0 (10.0, 10.0)
Ratio of PUFA and SFA	≥1	2.03 (1.22, 2.85)
Calcium (mg/d)	≥1,200	4.65 (3.19, 6.53)
Folate (mcg/d)	≥600	3.63 (2.39, 5.81)
Iron (mg/d)	≥27	5.46 (3.80, 7.44)
Total Score	90	47.4 (39.7, 56.5)

AHEI-P, Alternate Healthy Eating Index for Pregnancy; PUFA, Polyunsaturated Fatty Acid; SFA, Saturated Fatty Acids.

^a^Scored intermediate intake proportionately 0 and 10.

^b^1 serving = 0.5 cup of vegetables (1 cup = 236.59 gram).

^c^1 serving = 1 medium piece of fruit or 0.5 cup equivalent (0.5 cup = 118.30 gram).

**Table 2:** Blood pressure and blood glucose of pregnant women in selected primary health center in Jakarta (n = 174).

Variable	Mean ± SD
Fasting blood glucose (mg/dL)	89.6 ± 12.6
Systolic blood pressure (mmHg)	109 ± 11.9
Diastolic blood pressure (mmHg)	69 ± 8.47

SD, standard deviation.

The relationship of potential covariates with blood glucose and blood pressure are shown in Tables 3 and 4. Blood glucose level was significantly higher among women who had history of diabetes mellitus (DM) (108.0 ± 27.1 vs. 89.9 ± 11.4; p < 0.001), GDM (107.0 ± 31.28 vs. 89.1 ± 11.45; p < 0.001), and higher MUAC (r = 0.16; p = 0.03). Higher systolic blood pressure was found among women who had a family history of hypertension (121.7 ± 10.8 vs. 108.1 ± 11.4; p < 0.001), previously experienced hypertension (111.7 ± 10.9 vs. 107.4 ±12.15; p = 0.02), and higher MUAC (r = 0.41; p<0.001). Women with history of hypertension (77.5 ± 39.7 vs 68.3 ± 33.4, p < 0.001) and higher MUAC (r = 0.42; p<0.001) had significantly higher diastolic blood pressure.

**Table 3:** Relationship of potential covariates and diet quality with blood glucose of pregnant women (n = 174).

Variable	n	%	Blood Glucose	*p*
			Mean ± SD	
Age (years)				
< 20	11	6.3	90.6 ± 12.0	0.96^a^
20–34	132	75.9	89.5 ± 13.3	
≥ 35	31	17.8	89.5 ± 9.4	
Education (years)				
<9	45	25.9	87.1 ± 9.4	0.13^b^
≥9	129	74.1	90.4 ± 13.5	
Job				
Unemployed	118	67.8	90.1 ± 13.5	0.40^b^
Working	56	32.2	88.4 ± 10.6	
Parity				
Nulliparity	67	38.5	89.5 ± 13.3	0.94^b^
Multiparity	107	61.5	89.6 ± 12.2	
Smoking status				
Never	156	89.7	90.0 ± 12.9	0.15^b^
Past	18	10.3	85.5 ± 8.4	
MUAC	174	100	0.16^c^	0.03^d^*
Gestational Age	174	100	0.12^c^	0.11^d^
History of DM				
No	168	96.6	89.9 ± 11.4	<0.001**
Yes	6	3.4	108.0 ± 27.1	
History GDM				
No	169	97.1	89.1 ± 11.5	<0.001**
Yes	5	2.9	107.0 ± 31.3	
Family history of type 1 or 2 DM				
No	132	75.9	89.2 ± 12.6	0.50^b^
Yes	42	24.1	90.7 ± 12.8	
History fetal macrosomia				
No	167	96.0	89.4 ± 12.8	0.45^b^
Yes	7	4.0	93.1 ± 8.2	

SD, Standard Deviation; MUAC, Mid-Upper Arm Circumference; DM, Diabetes Mellitus; GDM, Gestational Diabetes Mellitus.

^a^ One–way ANOVA.

^b^ Independent *t-test*.

^c^ Correlation coefficient.

^d^ Pearson correlation.

*p<0.05.

**p<0.001.

**Table 4:** Relationship of potential covariates and diet quality with blood pressure of pregnant women (n = 174).

Variable	n	%	Systolic Blood Pressure	*p*	Diastolic Blood Pressure	*p*
			Mean ± SD		Mean ± SD	
Age						
< 20 years old	11	6.3	105.0 ± 11.9	0.44^a^	65.4 ± 30.2	0.23^a^
20–34 years old	132	75.9	109.0 ± 12.2		68.9 ± 35.3	
≥ 35 years old	31	17.8	110.4 ± 10.5		70.6 ± 13.7	
Education						
<9 years	45	25.9	108.8 ± 11.1	0.92^b^	67.4 ± 33.4	0.15^b^
≥9 years	129	74.1	109.1 ± 12.2		69.5 ± 34.9	
Job						
Unemployed	118	67.8	109.6 ± 12.0	0.36^b^	69.5 ± 10.9	0.28^b^
Working	56	32.2	107.8 ± 11.6		67.9 ± 34.1	
Parity						
Nulliparity	67	38.5	108.5 ± 12.1	0.68^b^	69.8 ± 33.1	0.78^b^
Multiparity	107	61.5	109.3 ± 11.8		69.1 ± 35.5	
Smoking status						
Never	156	89.7	108.6 ± 11.6	0.16^b^	68.9 ± 34.6	0.96^b^
Past	18	10.3	112.7 ± 14.3		69.1 ± 35.2	
MUAC	174	100	0.41^c^	<0.001^d^**	0.42^c^	<0.001^d^**
Gestational Age	174	100	0.05^c^	0.97^d^	0.03^c^	0.71^d^
History of Hypertension						
No	162	93.1	108.1 ± 11.4	<0.001^c^**	68.3 ± 33.4	<0.001^c^**
Yes	12	6.9	121.7 ± 10.8		77.5 ± 39.7	
Family history of ypertension						
No	109	62.3	107.4 ± 12.1	0.02^b^*	68.1 ± 35.9	0.09^b^
Yes	65	37.4	111.7 ± 10.9		70.4 ± 31.7	

SD, Standard Deviation; MUAC, Mid-Upper Arm Circumference.

^a^ One–way ANOVA.

^b^ Independent *t-test*.

^c^ Correlation coefficient.

^d^ Pearson correlation.

*p<0.05.

**p<0.001.

Table 5 shows the multivariate analyses of blood glucose and blood pressure. The middle tertile of AHEI-P score was associated with a 0.4 SD lower diastolic blood pressure (standardized effect size, 95% CI -0.7 to -0.06; p = 0.02) compared with the lowest tertile, after adjustment for level of education, smoking status, MUAC, gestatioal age, history of hypertension, and family history of hypertension. However, no association was found between the AHEI-P score and systolic blood pressure. Similarly, we did not find any significant associations between AHEI-P tertile and blood glucose after adjustment for level of education, smoking status, MUAC, gestational age, and personal or family history of DM.

**Table 5:** Multiple linear regression of diet quality with blood glucose and blood pressure among pregnant women in Jakarta (n = 174).

Variables	Effect size^a^	95% CI	*p*
**Blood Glucose**			
AHEI-P score	0.1	-0.09–0.6	0.33
<39.59	ref		
39.59–56.58	0.2	-0.2–0.6	0.32
>56.58	0.2	-0.2–0.5	0.33
≥9 years of education	0.2	-0.08–0.6	0.14
Ever-smoker	-0.5	-0.9–−0.003	0.05
MUAC	0.04	-0.02–0.07	0.07
Gestational age	-0.03	-0.07–0.0007	0.06
History of DM	1.5	0.7–2.2	<0.001**
Family history of DM	-0.02	-0.3–0.3	0.90
**Systolic Blood Pressure**			
AHEI-P score	0.1	-0.07–0.3	0.21
<39.59	ref		
39.59–56.58	-0.09	-0.4–0.2	0.57
>56.58	0.2	-0.2–0.6	0.37
≥9 years of education	-0.08	-0.4–0.2	0.68
Ever-smoker	0.2	-0.2–0.7	0.29
MUAC	0.1	0.07–0.2	<0.001**
Gestational age	0.01	-0.02–0.04	0.41
History of hypertension	0.8	0.08–1.5	0.03*
Family history of hypertension	0.3	-0.2–0.4	0.48
**Diastolic Blood Pressure**			
AHEI-P score	-0.09	-0.3–0.1	0.36
<39.59	ref		
39.59–56.58	-0.4	-0.7–−0.06	0.02*
>56.58	-0.2	-0.5–0.2	0.38
≥9 years of education	0.2	-0.09–0.5	0.17
Ever-smoker	-0.07	-0.5–0.3	0.73
MUAC	0.002	0.002–0.003	<0.001**
Gestational age	0.0003	-0.0005–0.001	0.48
History of hypertension	0.7	0.2–1.2	0.007*
Family history of hypertension	0.2	-0.1–0.4	0.23

CI, Confident Interval; MUAC, Mid-Upper Arm Circumference; AHEI-P, Alternate Healthy Eating Index for Pregnancy; DM, Diabetes Mellitus; GDM, Gestational Diabetes Mellitus.

^a^Standardized effect size.

*p<0.05.

**p<0.001.

## Discussion

This study indicates that higher diet quality among pregnant women was negatively associated with diastolic blood pressure. Mothers in the middle tertile of the AHEI-P score showed lower diastolic blood pressure by 0.4 SD (standardized effect size, 95% CI -0.7 to -0.06) compared to the lowest tertile. However, there were no significant associations between the diet quality with systolic blood pressure nor blood glucose. Overall, pregnant women had poor diet quality measured by AHEI-P and had systolic blood pressure ranging from 72 to 139 mmHg and diastolic blood pressure ranging from 51 to 98 mmHg, which is comparable to those in other LMICs [23, 24].

The assessment of dietary quality, blood glucose, and blood pressure was performed with proper procedures by trained and standardized field enumerators. To obtain a more accurate estimation of the actual food portion size, we used a food-photograph book that had been formulated for the large scale national food consumption survey from the Indonesian Ministry of Health [16]. For blood pressure assessment, we used a calibrated HBP-1300 Professional Blood Pressure device clinically proven to produce fast and reliable results [18]. The use of this digital device also reduced observer bias [25–27]. Fasting capillary blood glucose has been considered an acceptable and useful screening test for GDM [17]. As such, all assessments were reliable and within the known normal range for pregnant women. Importantly, while most studies have focused on the third trimester, our study provides data on the second trimester of pregnancy with outcomes suitable for this period.

Many studies have used the assessment of diet quality to investigate the role of complex dietary patterns on health risks [8, 19, 20, 28]. Diet quality was evaluated by AHEI-P, which was modified from the AHEI to enable integration with nutrition during pregnancy [8, 20]. Among the nine components of AHEI-P in this study, the median score of the ratio PUFA and SFA consumption was the lowest. Moreover, the median score for fruit and vegetable consumption was far from the recommended score, and much lower compared to another study in the United States [20]. The association between diet quality and diastolic blood pressure in our study is in line with the findings from previous research showing higher AHEI-P was associated with lower blood pressure [20].

Similarly, higher intake of fruits, vegetables, high unsaturated-to-saturated fat ratio, which were reflected in the DASH and Mediterranean diet and evaluated by AHEI-P, was associated with a lower risk of NCDs [29, 30]. Interaction of these food components would provide a better sodium-to-potassium ratio affecting plasma renin activity and lead to an increase of sodium excretion by the kidneys [31]. The essential nutrients, which are available from fruits and vegetables, are dietary fiber, magnesium, potassium, and calcium, which have been observed to provide anti-inflammatory and antioxidant activity and lower the risk of cardiovascular diseases [31–34]. Potassium, in particular, has demonstrated endothelium-dependent vascular effects that may lower blood pressure [35, 36].

Although AHEI-P was introduced in 2005, studies investigating its associations are limited, making it difficult to compare this study to others. It is known that physiological changes during pregnancy may influence blood pressure. The association with diet quality in this study was more pronounced for diastolic instead of systolic blood pressure. We note that systolic blood pressure is generally less sensitive than diastolic to risk factors during pregnancy, in part, due to increased cardiac output that compensates systemic vasodilatation in pregnancy. Therefore, systolic pressure might be less affected than diastolic [37].

Our study did not observe any significant association between diet quality and blood glucose. In contrast, a large cohort study by Sheryl et al. found that a 5-points increase of AHEI-P lowered blood glucose by 0.64 mg/dL [20]. Nonetheless, in line with our findings, a longitudinal study among pregnant women demonstrated no association between diet quality and blood glucose [38]. While a high intake of sugar-sweetened beverages has consistently been associated with a higher risk of type 2 diabetes, the AHEI-P index did not precisely capture dietary components related to carbohydrate, sugar-sweetened beverages, and added sugar [39, 40]. Additionally, pre-pregnancy overweight is an important predictor of GDM [41]. With these conditions, effects on insulin resistance would be more pronounced, and dietary factors could exhibit a greater impact on blood glucose concentration [42]. Unfortunately, our study could not provide data on pre-pregnancy BMI. Therefore, further studies with a larger sample size are required to confirm the finding on blood glucose.

To minimize potential under- or overestimation of nutrient content and to complement sparse data in Indonesian food composition tables for some nutrients, e.g. trans-fat, PUFA and SFA, we adopted the food composition database from other countries, e.g. the USA and Singapore, during dietary analysis. The AHEI-P index is based on the USDA guideline, which nearly parallels Indonesian recommendations [43]. This allowed us to apply the existing AHEI-P to the Indonesian context. Another study from Indonesia used the Mean Adequate Ratio (MAR) as the indicator for diet quality [44]; however, this was less relevant to our current research. The AHEI-P has also been implemented in countries outside the United States with or without adaptation, with generally reliable results [45–47].

A few limitations need to be considered to interpret our findings. In estimating the minimum sample size, we assumed a Spearman’s correlation of coefficient of 0.3. However, based on the analysis, the correlations were weaker than the expected, which suggests a larger sample size is required to confirm the findings. The poor-quality diet observed in pregnant women underscores the urgent need for the government to revitalize specific dietary guidelines for pregnant women. These should emphasize dietary patterns and diet quality in addition to quantity and single nutrients. Until now, WHO recommends that mothers should eat a variety of foods, including green and orange vegetables, meat, fish, beans, nuts, whole grains, and fruits, in addition to the iron, folate, and calcium supplementation, and with the option to utilize multiple micronutrient supplementation [48]. This recommendation can serve as a starting point for the local context. Influences from the environment, e.g. home food environment, also need to be taken into account to improve the adherence of the mothers to the dietary guideline, and in turn may decrease the prevalence of NCDs in pregnancy, and reduce maternal and newborn death and complicated birth outcomes [48–50]. Since mothers are now facing an unprecedented situation in the COVID-19 pandemic, there is an even greater need to enhance access to healthy foods. In addition, other habits related to hypertension, such as physical activity and smoking cessation, are necessary to be integrated into the recommendations.

Our study highlights the importance of diet improvement to reduce the risk of NCDs in this population of urban women from an Asian megacity in a LMIC, especially during the pre-pregnancy period. Because perturbations in pregnancy might affect fetal growth and development, additional studies are warranted to investigate the impact of diet quality on fetal outcomes, such as birth weight, prematurity, and mortality. Furthermore, future research is recommended to explore the most effective integrated package of interventions to reduce the risk of NCDs in pregnancy.

## Conclusions

Higher dietary quality was negatively associated with lower diastolic blood pressure among pregnant women in the Asian megacity of Jakarta, Indonesia, but did not associate with systolic blood pressure and blood glucose levels. Behavior changes in pregnancy need to address the importance of multiple inadequate dietary patterns of specific food components, including PUFA, fruits, vegetables. An innovative intervention is needed with integrated dietary improvement and biomarker tracking using interactive digital tools, diet, and psychosocial support linked with high-performing frontline health workers to optimize blood glucose and blood pressure in pregnancy and pre-pregnancy, and improve maternal and fetal outcomes [51].

## Supporting information

S1 File (XLSX)
